# A haplotype-resolved genome assembly of *Rhododendron vialii* based on PacBio HiFi reads and Hi-C data

**DOI:** 10.1038/s41597-023-02362-1

**Published:** 2023-07-12

**Authors:** Yuhang Chang, Rengang Zhang, Yongpeng Ma, Weibang Sun

**Affiliations:** 1 Yunnan Key Laboratory for Integrative Conservation of Plant Species with Extremely Small Populations, Kunming Institute of Botany, Chinese Academy of Sciences, Kunming, 650201 China; 2 Key Laboratory for Plant Diversity and Biogeography of East Asia, Chinese Academy of Sciences, Kunming Institute of Botany, Kunming, 650201 China; 3 University of Chinese Academy of Sciences, Beijing, 101408 China; 4 Kunming Botanical Garden, Kunming Institute of Botany, Chinese Academy of Sciences, Kunming, 650201 China

**Keywords:** Plant molecular biology, Genome

## Abstract

*Rhododendron vialii* (subgen. *Azaleastrum*) is an evergreen shrub with high ornamental value. This species has been listed as a plant species with extremely small populations (PSESP) for urgent protection by China’s Yunnan provincial government in 2021, due to anthropogenic habitat fragmentation. However, limited genomic resources hinder scientifically understanding of genetic threats that the species is currently facing. In this study, we assembled a high-quality haplotype-resolved genome of *R*. *vialii* based on PacBio HiFi long reads and Hi-C reads. The assembly contains two haploid genomes with sizes 532.73 Mb and 521.98 Mb, with contig N50 length of 35.67 Mb and 34.70 Mb, respectively. About 99.92% of the assembled sequences could be anchored to 26 pseudochromosomes, and 14 gapless assembled chromosomes were included in this assembly. Additionally, 60,926 protein-coding genes were identified, of which 93.82% were functionally annotated. This is the first reported genome of *R*. *vialii*, and hopefully it will lay the foundations for further research into the conservation genomics and horticultural domestication of this ornamentally important species.

## Background & Summary

*Rhododendron* L. is the largest genus in the Ericaceae, and is well known for its ornamental value, ecological significance and cultural importance. More than 600 species of *Rhododendron* have been recorded from China^1,2^, making it the largest genus of seed plants in the country^3^. However, despite the high *Rhododendron* species diversity in China, many *Rhododendron* species have recently been threatened due to climate change, habitat loss and disturbance from human activities^4,5^. According to *The Red List of Rhododendrons*, of the 665 taxa (including infraspecific taxa) native to China, 15 species are Critically Endangered (CR), 18 are Endangered (EN) and 180 are Vulnerable (VU). These threatened species account for 32.03% of *Rhododendron* species diversity in China, and there are further 183 species for which there are insufficient data to assess their conservation status (Data Deficient, DD) meaning that this genus is in urgent need of detailed investigation and conservation in China^6^.

**Fig. 1:**
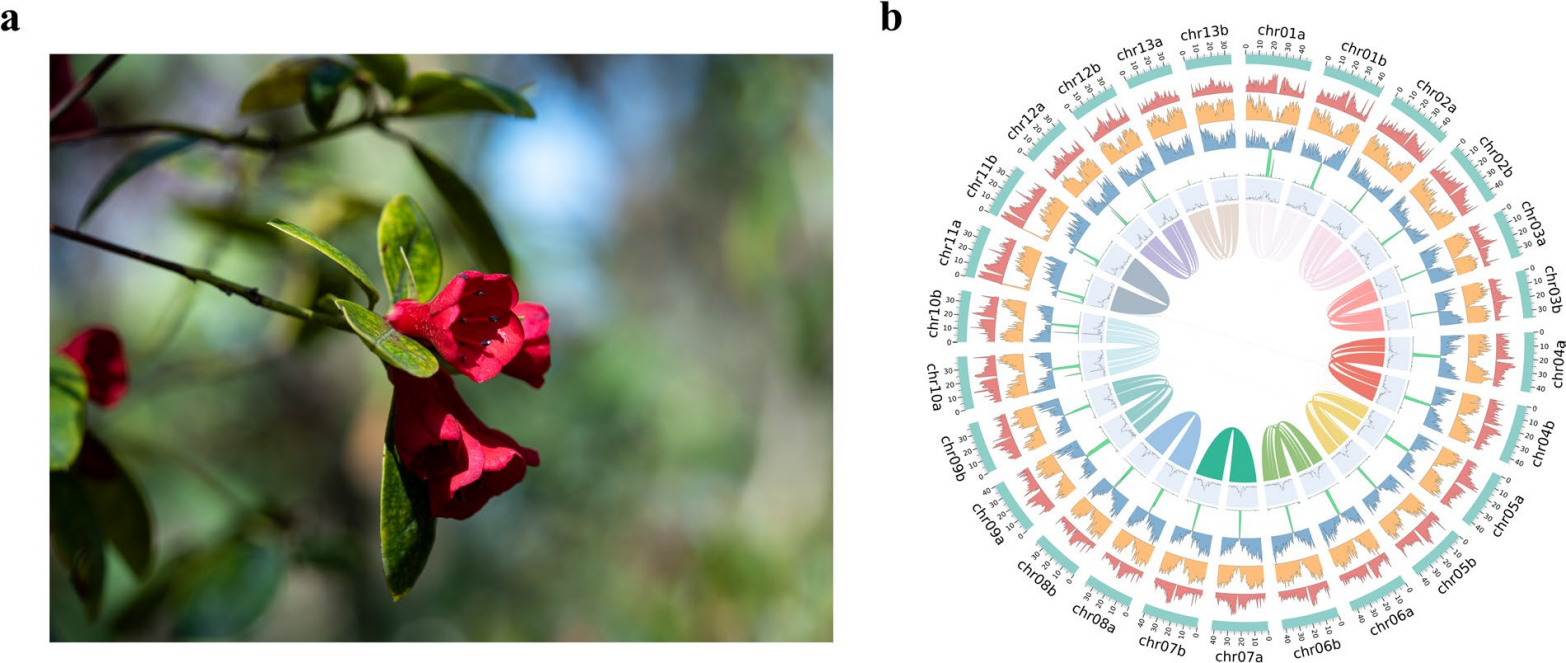
Morphological characters (**a**) and features of the haplotype-resolved genome assembly (**b**) of *R*. *vialii*. From the outer ring to the inner ring are the distributions of pseudochromosomes, class I TE density, class II TE density, protein-coding gene density, proportion of tandem repeat, GC content and syntenic blocks.

*Rhododendron vialii* Delavay & Franch. is an evergreen shrub belonging to the subgenus *Azaleastrum* Planch. ex K. Koch (Fig. 1a), and it has the only red tubular-funnelform corolla of this subgenus. According to specimen records, *R*. *vialii* was once extremely abundant, but has become gradually rarer in recent years due to deforestation, overcollection and habitat destruction^7^. *R*. *vialii* was evaluated as VU in *The Red List of Rhododendrons*^6^, and it is also included on the 2021 edition of the *List of Yunnan Protected Plant Species with Extremely Small Populations (PSESP)*, which is part of a project for rescuing the most threatened plant species in China^8,9^.

In this study, we sampled an *ex-situ* conserved *R*. *vialii* and successfully assembled a high-quality haplotype-resolved, nearly telomere-to-telomere genome by combining PacBio high-fidelity (HiFi) reads and high-throughput chromosome conformation capture sequencing (Hi-C) reads. The final assembled genome contains haplotype A (532.73 Mb) and haplotype B (521.98 Mb), with contig N50 length of 35.67 Mb and 34.70 Mb, respectively (Fig. 1b). Based on the karyotype (2n = 26)^10^, approximately 99.92% of the assembled data was anchored to pseudochromosomes, and our final assembly contains 14 gapless assembled chromosomes.

We ran a Benchmarking Universal Single-Copy Orthologs assessment (BUSCO^11^, v. 5.3.2) with the lineage dataset embryophyta_odb10. The complete BUSCOs (including single-copy and multi-copy) of the two haplotypes accounted for 98.5% and 98.1%, respectively, indicating good completeness of the genome. In addition, we annotated 551.06 Mb (52.19% of the whole genome) corresponding to repeat elements. A total of 60,926 protein-coding genes were identified, of which 93.82% could be functionally annotated. This is the first report of a *R*. *vialii* genome sequence, and we believe that it will provide an important resource allowing us to explore the mechanisms underlying threats to this species, as well as its evolutionary history and further utilization on ornamental horticulture.

## Methods

### Sampling

For genomic DNA extraction, fresh young leaves of *R*. *vialii* were collected from a single adult plant in Kunming Botanical Garden, Kunming Institute of Botany, Chinese Academy of Sciences. We also collected roots, branches, leaves, buds and fruits for transcriptome sequencing. These materials were frozen directly in liquid nitrogen and were then transferred to –80°C for preservation. The related sequencing services were performed by a commercial sequencing provider (Wuhan Benagen Technology Co. Ltd. Wuhan, China).

### Genome sequencing

A modified CTAB methods was performed to extract total DNA from young *R*. *vialii* leaves^12^. The concentration of DNA was assessed using NanoDrop (NanoDrop Technologies, Wilmington, DE, USA) and a Qubit 3.0 fluorometer (Life Technologies, Carlsbad, CA, USA). 1% agarose gel electrophoresis was then used to assess the purity and integrity of the resulting DNA. The short-read library with a DNA-fragment insert size of 200–400 bp was prepared using 1 μg genomic DNA following the manufacturer’s instructions (BGI) and was subject to paired-end (PE) sequencing on a DNBSEQ-T7 platform (BGI lnc., Shenzhen, China) using a PE 150 model. This produced 97.39 Gb (~649 M reads) of raw data, meaning approximately 150× genome coverage (Supplementary Table 1).

Before long-read sequencing, the DNA was purified using a DNeasy Plant Mini Kit (Qiagen, Germantown, MD, USA), and the integrity of the DNA was evaluated with a Femto Pulse (Agilent Technologies, Santa Clara, CA, USA). Megaruptor 3 (Diagenode SA., Seraing, Belgium) was used to shear 8 μg genomic DNA, and these fragments were then concentrated using AMPure PB magnetic beads (Pacific Biosciences, Menlo Park, CA, USA). Each PacBio single molecule real-time (SMRT) library was prepared using a SMRT bell express template prep Kit 2.0 (Pacific Biosciences, Menlo Park, CA, USA), with insert sizes of 15 kb selected using BluePippin system (Sage Science, Beverly, MA, USA). The library was sequenced on a Pacific Bioscience Sequel II platform in CCS mode, and the raw data was converted into high-precision HiFi reads using the CCS workflow^13^ (v. 6.3.0) with the standard parameters. From this process, we obtained 32.88 Gb (~60×) of HiFi data with an average read length and N50 read length of about 18 kb (Supplementary Table 2).

### Hi-C library construction and sequencing

Hi-C libraries were prepared following a modified protocol from Belton *et al*.^14^. Fresh leaf tissue was fixed in 2% formaldehyde solution, and the cross-linked DNA was then digested with DpnII. Biotin-labeled adapters were attached at the sticky ends of the digested fragments to form chimeric junctions, which were enriched and trimmed to fragments of about 450 bp for pair-end sequencing on a DNBSEQ-T7 platform. Approximately 75 Gb (~500 M reads) of Hi-C data was generated for subsequent pseudochromosome assist assembly (Supplementary Table 3).

### Transcriptome sequencing

Materials for transcriptome sequencing were homogenized and total RNA was extracted using R6827 Plant RNA Kit (Omega Bio-Tek, Norcross, GA, USA) following the manufacturer’s instructions. Subsequently, SQK-PCS109 and SQK-PBK004 Kits (Oxford Nanopore Technologies, Oxford, UK) were combined to prepare the library, and the library was sequenced using a Nanopore PromethION sequencer. Finally, a total of 10.36 Gb (~10.89 M reads) full-length RNA-seq data were obtained for genome annotation (Supplementary Table 4).

### Preliminary genome survey

The preprocessor, fastp^15^ (v. 0.19.3) was used to filter out the adapter sequences, overly short reads and low-quality reads from the next generation sequencing data using the default parameters. Jellyfish^16^ (v. 2.2.10) was then used to calculate the frequency distribution of the depth of clean data with 19-mers, and the basic features of the genome were estimated with the software GCE^17^ (v. 1.0.0). The estimated genome size of *R*. *vialii* is about 525.63 Mb, with a heterozygosity of 0.89% and a duplication rate of 43.47% (Supplementary Table 5 and Supplementary Fig. 1).

### Chromosome-level genome assembly

HiFi reads and Hi-C short reads were used as a combined input for the genome assembler Hifiasm^18^ (v. 0.16.1-r375) and were assembled into a pair of haplotype-resolved assembly contigs (haplotype A and haplotype B) in Hi-C mode with the default parameters. Juicer^19^ (v. 1.5.6) was then used to map clean Hi-C reads to the contigs, and Hi-C-assisted initial chromosome assembly was conducted using the 3D-DNA^20^ (v. 180922) algorithm with the standard procedure. Chromosome boundaries were adjusted and the scaffold corrected using the manually operated Juicebox^21^ (v. 1.11.08) module, and the generated file was used as input for 3D-DNA for re-scaffolding by chromosome. Juicebox was used again for re-quality control and adjustment of mis-joins and orientation to generate chromosome scaffolds and un-anchored sequences. Additionally, TGS-GapCloser^22^ (v. 1.1.1) was employed to fill gaps of the genome based on the HiFi reads.

Because some of the telomere assemblies were incomplete or missing, Minimap2^23^ (v. 2.24-r1122) was used to map the HiFi reads to the chromosome, and the reads aligned to the positions of the telomeres were assembled into contigs using Hifiasm. These contigs were then mapped to the chromosomes to extend the chromosomal ends. GetOrganelle^24^ (v. 1.7.5) was used to assemble the chloroplast and mitochondrial genomes. After the above steps were completed, the software Nextpolish^25^ (v. 1.3.1) was employed to polish the assembly based on the short reads from two iterations, and Redundans^26^ (v. 0.13c) was used to remove redundancies such as haplotigs and rDNA fragments. Overall, about 99.92% of the assembled data was anchored to pseudochromosomes in the two haplotypes (Supplementary Table 6), and the chromosome number was set based on the results of the karyotype analysis (2n = 26). Finally, we obtained a high-quality haplotype-resolved gapless genome of *R. vialii*.

The assembly (approx. 1.05 Gb) contained two complete haplotypes, haplotype A and haplotype B, with genome sizes of 532.73 Mb and 521.98 Mb, respectively (Table 1). The genome size previously estimated based on K-mers was similar to these assemblies, with the main deviations in rDNA array. The contig N50 length of haplotype A and haplotype B were 35.67 Mb and 34.70 Mb, respectively. The number of gaps was fewer than 10 in both haplotypes (Table 1), and 14 gapless chromosomes were assembled (Supplementary Table 6), indicating good continuity of assembly.

**Table 1:** Summary of the *Rhododendron vialii* genome assembly data.

Statistic	Haplotype A		Haplotype B	
Total size (bp)	532,733,691		521,982,192	
Number of gaps	5		7	
GC content (%)	39.52		39.43	
Characteristic	Contig	Scaffold	Contig	Scaffold
Number	18	13	20	13
Max. (bp)	49,061,468	49,061,468	44,137,505	46,758,727
Mean (bp)	29,596,288	40,979,514	26,099,074	40,152,476
Min. (bp)	11,008,751	34,773,050	9,515,951	34,698,656
Median (bp)	32,928,375	40,475,278	25,832,893	39,425,882
N10 (bp)	44,228,087	47,677,579	42,443,326	45,509,457
N50 (bp)	35,672,969	42,050,137	34,698,656	41,813,812
N90 (bp)	18,952,633	35,586,280	14,046,180	35,674,509
L10	2	2	2	2
L50	7	6	7	6
L90	14	12	16	12

### Identification of repetitive elements

EDTA^27^ (v. 1.9.9; parameters: --sensitive 1 --anno 1) was used for *de novo* identification of transposable elements to generate the TE library, and RepeatMasker^28^ (v. 4.0.7) was employed to detect repetitive elements in the assembled genome of *R*. *vialii* with the default parameters. We identified a total of 1,534,208 repetitive sequences (~ 551.06 Mb), accounting for 52.19% of the assembled genome, of which long terminal repeats (LTRs) and terminal inverted repeats (TIRs) had the highest proportions, accounting for 27.18% and 17.25% of the genome, respectively (Supplementary Table 7).

### Gene identification and functional annotation

Transcriptome assembly was based on multiple strategies: (i) next-generation RNA-seq data was downloaded from the National Center for Biotechnology Information (NCBI) Sequence Read Archive (SRA) database (SRR13338561)^29^ for *de novo* assembly using Trinity^30^ (v. 2.0.6); (ii) short-reads were aligned to the reference genome using Hisat2^31^ (v. 2.1.0) and then assembled using StringTie^32^ (v. 1.3.5); (iii) Minimap2 was used to map the long-reads to the genome and StringTie was employed for further assembly. Ultimately, PASA^33^ (v. 2.4.1) was used to combine and optimize the transcriptomes obtained by the above methods and to generate a high-quality transcriptome with a total length of 122.34 Mb and 114,558 transcripts (Supplementary Table 8). The publicly available combined 285,362 non-redundant protein sequences (including *R*. *griersonianum*^34^, *R*. *molle*^35^, *R*. *delavayi*^36^, *R*. *simsii*^37^, *R*. *williamsianum*^38^, *R*. *ovatum*^39^, *R*. *henanense* subsp. *lingbaoense*^40^, *Vaccinium macrocarpon*^41^, *Actinidia chinensis*^42^, *Camellia sinensis*^43^, *Camptotheca acuminata*^44^, *Coffea canephora*^45^ and *Vitis vinifera*^46^) were used as homologous protein evidence for this gene annotation. The PASA process was used to annotate the structure of the genomic genes based on transcript evidence, and the full-length genes were detected by comparison with reference proteins. The parametric model of AUGUSTUS^47^ (v. 3.4.0) was trained with the full-length gene set for five rounds of optimization.

The MAKER2^48^ (v. 2.31.9) pipeline was used for annotation based on *ab initio* prediction, transcript evidence and homologous protein evidence. Briefly, AUGUSTUS was used for *ab initio* protein-coding gene prediction after masking the repetitive regions of the genome with RepeatMasker. The transcripts were then aligned to the repeat-masked genome using BLASTN and TBLASTX, while BLASTX was employed to aligned protein sequences to the genome. The previous results were then optimized using Exonerate^49^ (v. 2.2.0) and the hints files were generated based on the results. Integration of the predicted gene models was conducted using AUGUSTUS and UTR annotations were added according to the EST evidence.

Considering the relatively low accuracy of annotation results from the MAKER2 process, we also used EVidenceModeler^50^ (EVM, v. 1.1.1) to integrate the predictive results obtained from the PASA and MAKER2 annotations. To avoid introducing TE coding regions, TEsorter^51^ (v. 1.4.1) was used to identify the TE protein domains on the genome and EVM was used to mask them. Additionally, PASA was used to optimize the results obtained by EVM, and UTR sequences and alternative splicing were added, and overly short and abnormal gene annotations were removed. For non-coding RNAs, we used tRNAScan-SE^52^ (v. 2.0.7) to identify the tRNAs and Barrnap (v. 0.9)^53^ to detect the rRNAs. RfamScan^54^ (v. 14.2) was used to annotate various non-coding RNAs. Finally, all annotation results were merged to remove the redundancy and a complete gene set was obtained.

Overall, a total of 60,926 protein-coding genes have been successfully predicted, with an average length of 5,378.1 bp. Among them, there were 79,618 coding sequences (CDS) and 460,225 exons, and the mean length was 1,275.7 bp and 328.4 bp, respectively (Table 2). In addition, we also identified 5,538 non-coding genes, which contained 3,039 rRNAs, 770 tRNAs and 1,729 other ncRNAs.

**Table 2:** Gene annotation statistics.

Feature	All genes					Coding genes				
	Number	Min.	Max.	Median	Mean	Number	Min.	Max.	Median	Mean
Gene	66,464	47	308,786	3,128	4,948.8	60,926	153	308,786	3,589	5,378.1
Transcript	85,156	47	18,500	1,565	1,789.8	79,618	153	18,500	1,656	1,898.7
CDS	79,618	153	17,796	1,059	1,275.7	79,618	153	17,796	1,059	1,275.7
Exon	465,832	3	16,278	156	327.2	460,255	3	16,278	158	328.4
Intron	380,676	7	196,915	383	886.1	380,637	21	196,915	383	886.1
Exons/Transcript	85,156	1	79	4	5.5	79,618	1	79	4	5.8

For the functional prediction of protein-coding genes, three strategies were used. The predicted genes were aligned with the eggNOG v. 5.0 homologous gene database using eggNOG-mapper^55^ (v. 2.0.1) for Gene Ontology (GO) and Kyoto Encyclopedia of Genes and Genomes (KEEG) annotation. The protein sequences were matched to the protein databases, including Swiss_Prot, TrEMBL, NR (non-redundant protein) and the Arabidopsis database using DIAMOND^56^ (v. 2.0.4; Identity > 30%, E-value < 1e-5) to determine the best alignment of the genes. To obtain the conserved amino acid sequences, motifs and domains of the predicted proteins, InterProScan^57^ (v. 5.14–53.0) was used to search for similarity of domain according to the sub-databases PRINTS, Pfam, SMART, PANTHER and CDD of the InterPro database. Finally, 57,225 genes were functionally annotated in at least one of the above databases, accounting for 93.82% of the predicted protein-coding genes (Supplementary Table 9).

### Chromosomal synteny analysis

Minimap2 was used to compare the assembly of *R. vialii* with the previously published *R*. *griersonianum* genome, which showed that they had essentially the same order of chromosomes (Supplementary Fig. 2a). Meanwhile, the dot-plot of syntenic blocks between the two haplotypes showed a similar result (Supplementary Fig. 2b).

To explore the differences between the two haplotypes, the NUCmer module embedded in MuMmer 4^58^ was used to align the whole genome, and the command delta-filter was used to remove short and low-quality alignments. After the subprogram show-coords was used for format conversion, we ran SyRI^59^ (v. 1.6) to detect variations. A total of 3,695 syntenic regions (~458 Mb) were detected, indicating high similarity between the two haplotypes. However, we also identified many variations, including 3,152,185 SNPs and 214,391 small insertions/deletions (indels, < 500 bp). We additionally identified 2,157 and 1,486 duplications in haplotypes A and B, respectively. Other differences between the two assemblies included 1,108 translocations and 48 inversions. Notably, two relatively large inversions of more than 1 Mb were found on Chr1 and Chr10, respectively (Supplementary Fig. 3).

## Data Records

The relevant data reported in this paper have been deposited in the National Genomics Data Center (NGDC)^60,61^, Beijing Institute of Genomics, Chinese Academy of Sciences/China National Center for Bioinformation, under the BioProject accession number PRJCA015878 that is publicly accessible at https://ngdc.cncb.ac.cn/gwh. BGI short-reads, PacBio HiFi long-reads, Hi-C reads and Iso-Seq data have been deposited in the Genome Sequence Archive (GSA) in NGDC under the accession number CRR719647^62^, CRR719646^63^, CRR719645^64^ and CRR719648^65^. The final chromosome assembly and annotation data were deposited in the Genome Warehouse (GWH) in NGDC under the accession number GWHCAXW00000000^66^. The sequence data were also deposited in the SRA database with accession number SRR24501948^67^ SRR24501949^68^ SRR24501947^69^ and SRR24501946^70^ under the BioProject accession number PRJNA971245. And the final genome assembly has also stored in the GenBank with accession number GCA_030253575.1^71^ and GCA_030253555.1^72^.

## Technical Validation

### Evaluation of the assembled genome

**Fig. 2:**
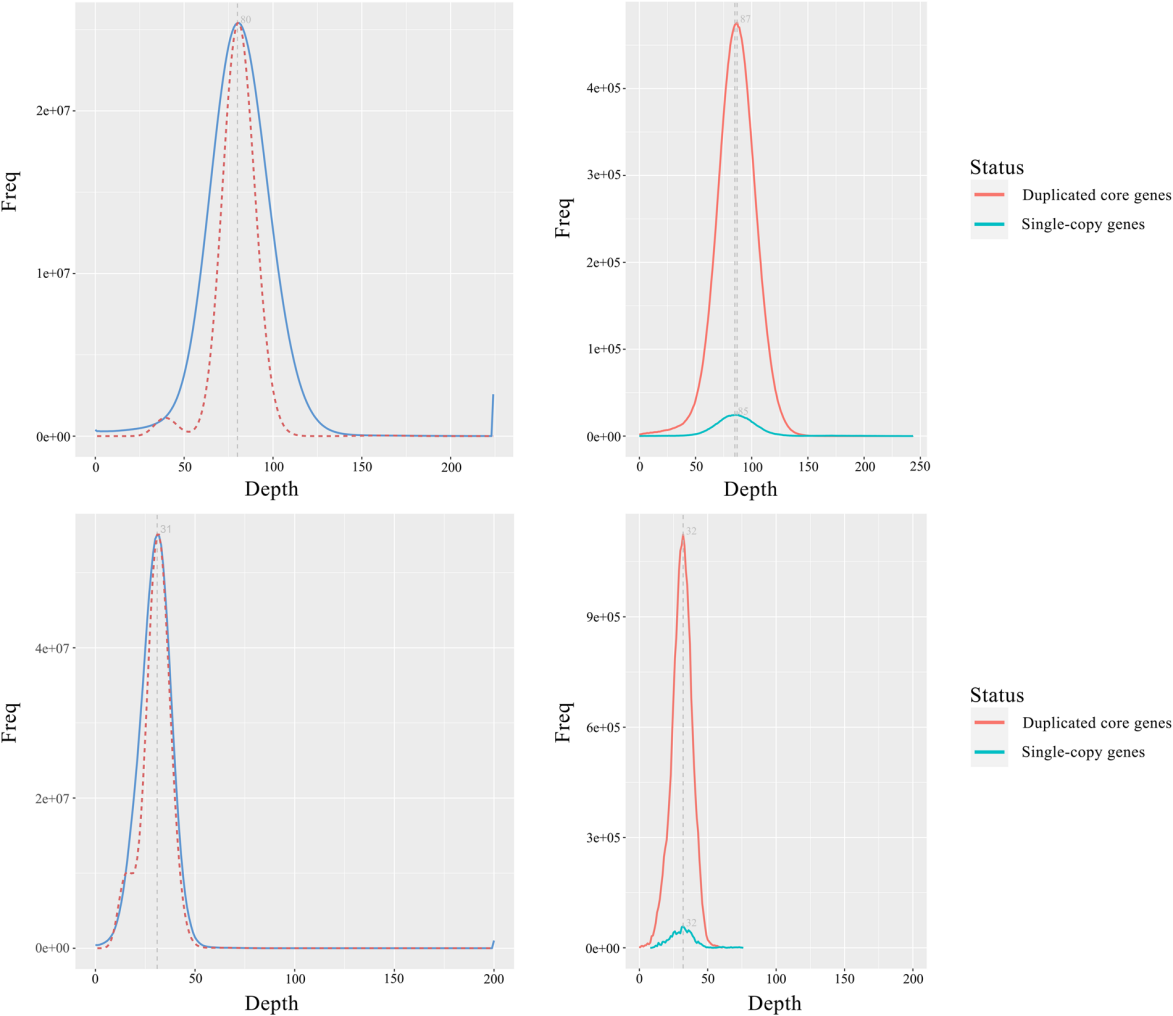
The distribution of coverage depth of the genome (left) and BUSCO core region (right) evaluated by the next-generation data (upper) and HiFi data (lower).

The BUSCO analysis with the lineage dataset embryophyta_odb10, which was conducted to assess genome completeness, showed that, for 1,614 expected genes from the embryophyta, the proportions of complete BUSCOs (including single-copy and multi-copy) of these two haplotypes were 98.5% and 98.1%, respectively, indicating good genomic completeness (Table 3). Short-reads and long-reads were mapped to the genome with BWA^73^ (v. 0.7.17-r1188) and Minimap2, and the short RNA-seq reads were mapped to the assembly using Hisat2. After filtering out non-primary alignments, the map ratio and coverage of reads were calculated. We found that different types of sequencing data had a high genome coverage (Table 4). The distribution of coverage depth of these sites on the genome matched the Poisson distribution and there was no obvious heterozygous peak. The BUSCO single-copy and multi-copy genes have approximately the same depth, indicating that the assembly had no redundancy (Fig. 2).

**Table 3:** Statistics of BUSCO evaluation of the two haplotypes and proteins.

Type	BUSCO groups		
	Haplotype A	Haplotype B	Proteins
Complete BUSCOs (C)	1,589 (98.5%)	1,584 (98.1%)	1,583 (98.1%)
Complete and single-copy BUSCOs (S)	1,522 (94.3%)	1,513 (93.7%)	69 (4.3%)
Complete and duplicated BUSCOs (D)	67 (4.2%)	71 (4.4%)	1,514 (93.8%)
Fragmented BUSCOs (F)	9 (0.6%)	8 (0.5%)	6 (0.4%)
Missing BUSCOs (M)	16 (0.9%)	22 (1.4%)	25 (1.5%)
Total BUSCO groups searched	1,614	1,614	1,614

**Table 4:** Statistics of map rate and coverage of different types of sequencing reads.

Data set	HiFi	Next generation	Iso-Seq	RNA-Seq
Reads mapped (%)	99.77	99.81	89.88	80.15
Properly paired (%)	—	84.11	—	75.44
Bases mapped (%)	99.74	99.83	93.29	80.41
≥1× (%)	99.96	99.96	22.67	11.86
≥5× (%)	99.76	99.85	9.82	6.07
≥10× (%)	99.12	99.71	7.08	4.34
≥20× (%)	90.00	99.38	4.99	2.96

**Fig. 3:**
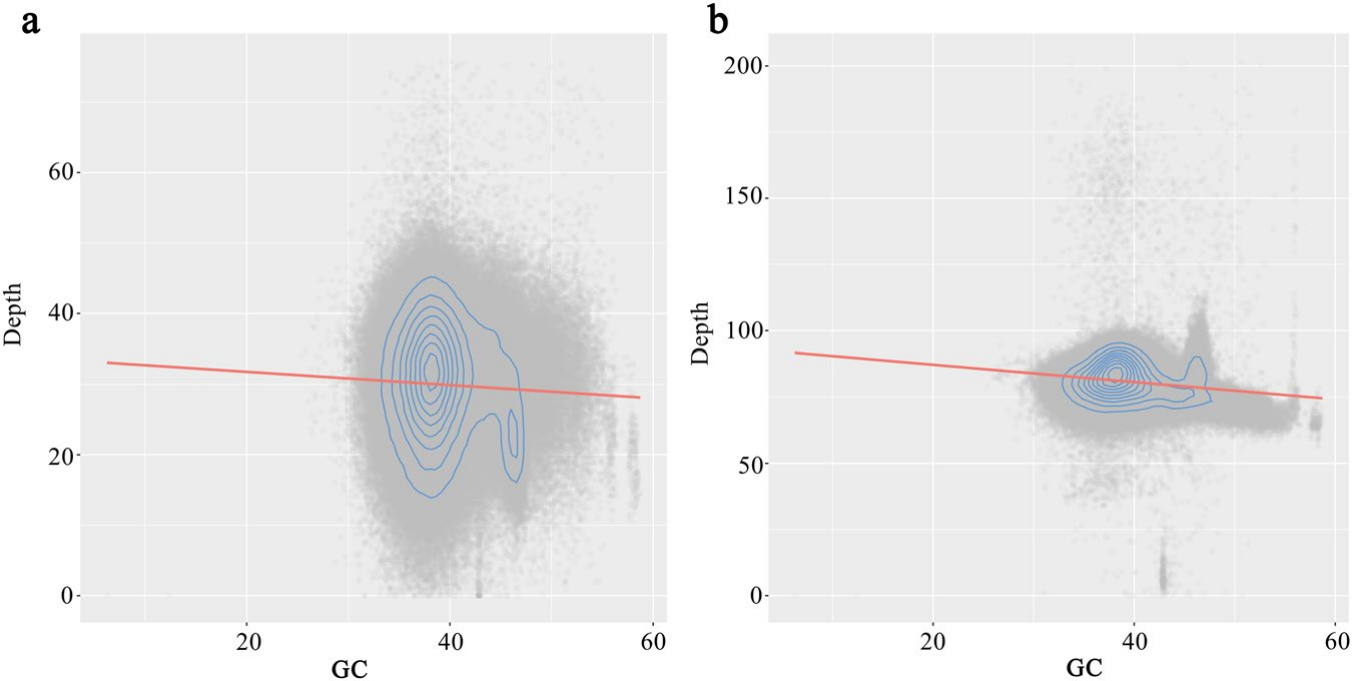
Coverage depth distribution of HiFi data (**a**) and the next-generation data (**b**).

**Fig. 4:**
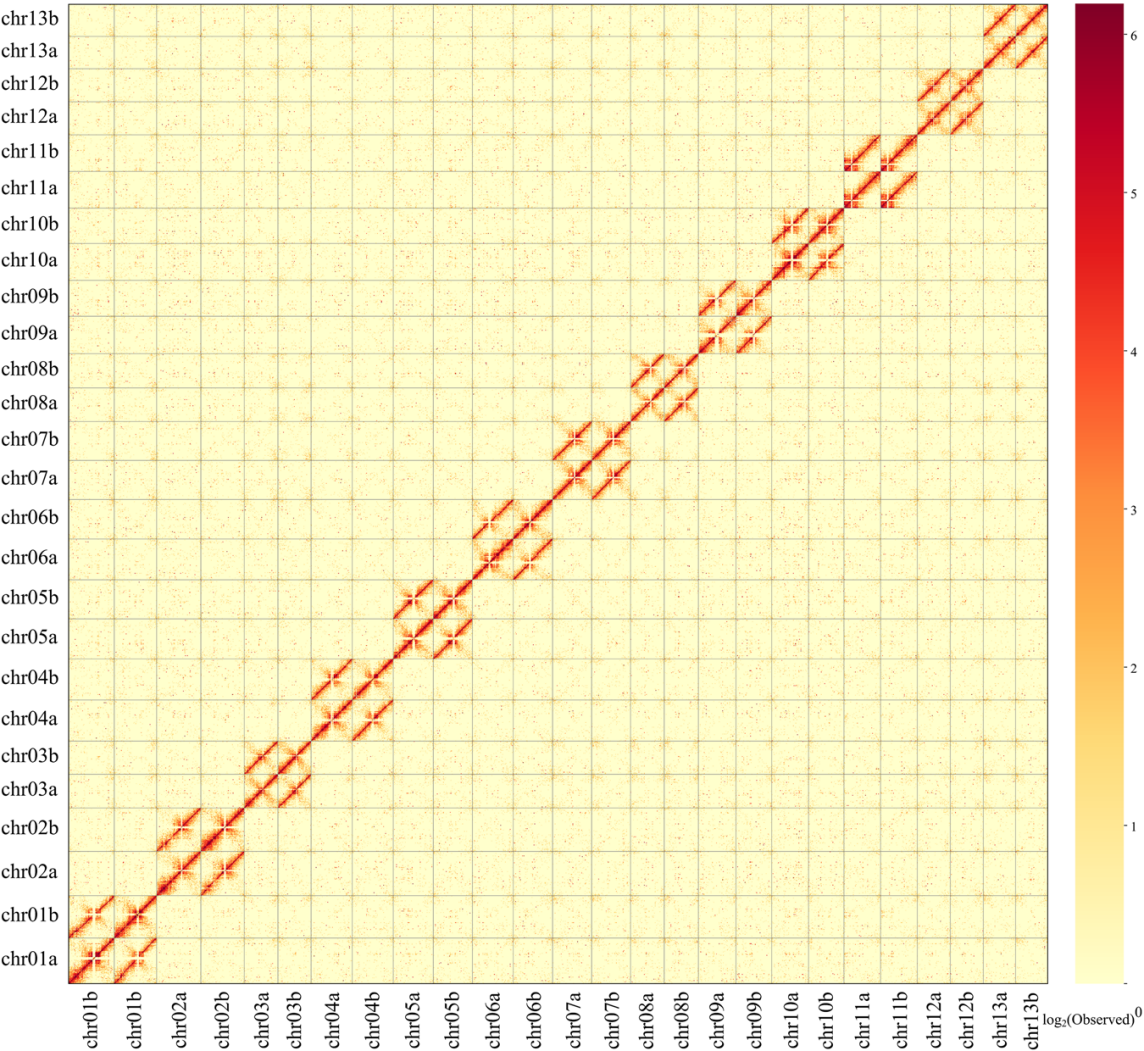
Hi-C interactive heatmap (with a resolution of 100 kb and the minimum mapping quality was one) of the two haploids. The strength of the interaction was represented by the color from yellow (low) to red (high).

To assess single base error rate and heterozygosity, the next-generation reads were mapped to the genome using BWA, and the upstream data was input to bcftools^74^ (v. 1.11) for variant detection. The calculated heterozygosity was approximately 0.0038% based on the heterozygous sites, and the single base error rate was about 0.00021% based on the homozygous loci. There was no obvious guanine-cytosine (GC) bias in the coverage depth analysis based on second and third generation data under different GC content (Fig. 3). Hi-C reads were mapped to the final version of the assembly using Juicer. In the Hi-C heatmap, there were strong interactive signals of the 13 chromosomes around the diagonal, suggesting that the two assemblies were without obvious chromosome assembly errors (Fig. 4).

**Fig. 5:**
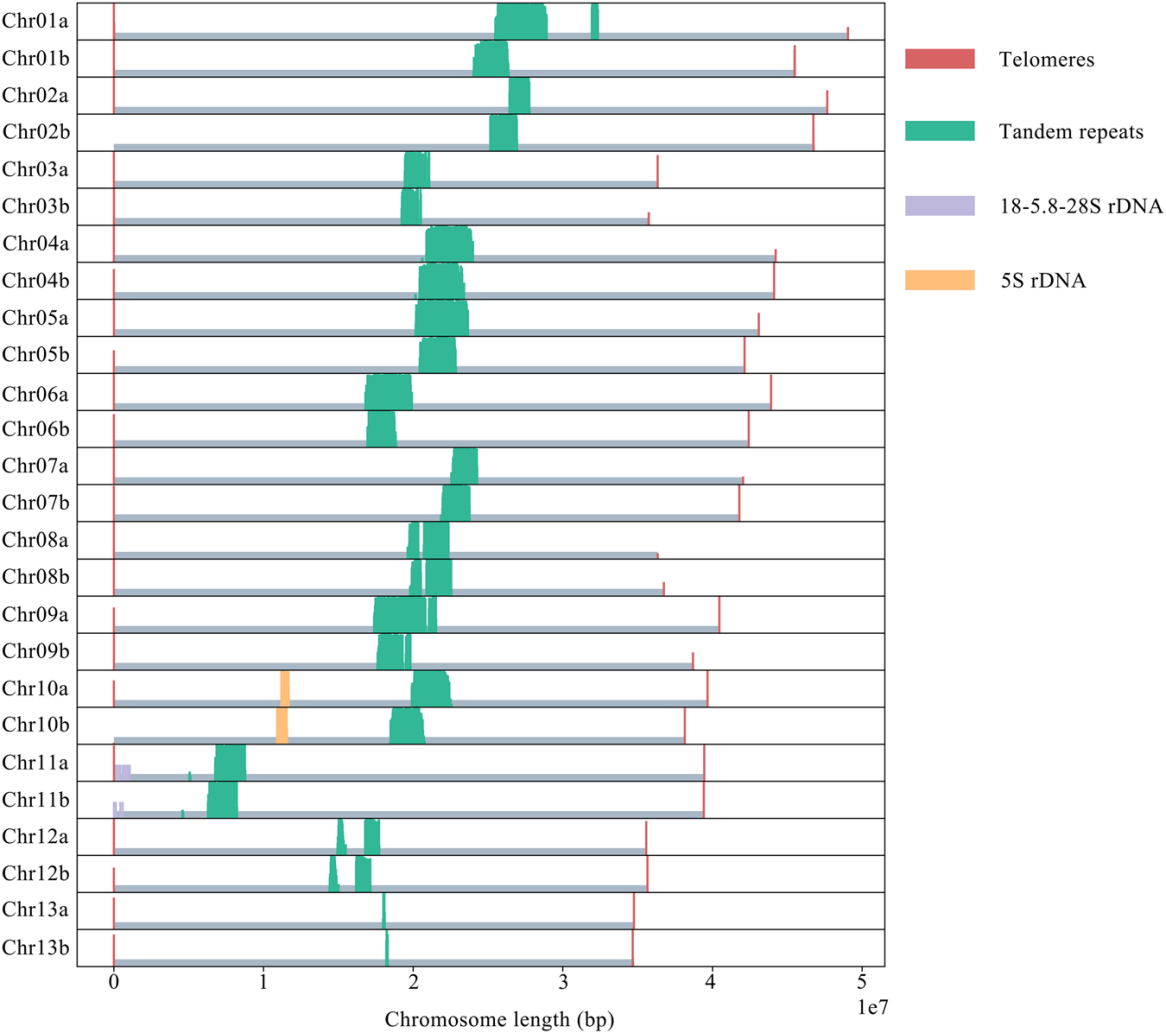
Bar-plot of density and distribution of several repeated elements on chromosome sequences of *R*. *vialii*.

The repetitive sequences identified by RepeatMasker were mapped to the genome to determine the position of the telomeres and other characteristic sequences on the chromosomes. Most of the chromosomes assembled complete telomere sequences (TTTAGGG), and only a few telomeres were missing or incomplete. All chromosomes contained a high tandem repeat (TGGTACCGTATGGATGTACTCGTACGGTATTGTACCGTTTTGGTGTGGTT), which is probably the centromere. In addition, the 18-5.8-28S rDNA and 5S rDNA arrays were detected on Chr11 and Chr10 respectively (Fig. 5). In summary, this assembly can by described as a nearly telomere-to-telomere genome.

### Evaluation of the gene annotation

The annotated and integrated proteins were also evaluated using BUSCO with the lineage dataset embryophyta_odb10. Briefly the proportion of complete core gene coverage was 98.1% (including 4.3% single-copy genes and 93.8% duplicated genes) and there were only a few fragmented (0.4%) and missing (1.5%) genes (Table 3), indicating that this annotation was of high quality.

## Supplementary information

Supplementary Table

Supplementary Figure

## Data Availability

All software and pipelines were executed according to the manual and protocols of the published bioinformatic tools. The version and code/parameters of the software have been described in the Methods section.

## References

[1] Tian X, Chang Y, Neilsen J, Wang S, Ma Y (2019). A new species of *Rhododendron* (Ericaceae) from northeastern Yunnan. China. Phytotaxa.

[2] Chang Y (2021). *Rhododendron kuomeianum* (Ericaceae), a new species from northeastern Yunnan (China), based on morphological and genomic data. Plant Divers..

[3] Yan L (2015). DNA barcoding of *Rhododendron* (Ericaceae), the largest Chinese plant genus in biodiversity hotspots of the Himalaya-Hengduan mountains. Mol. Ecol. Res..

[4] Ma Y, Nielsen J, Chamberlain DF, Li X, Sun W (2014). The conservation of *Rhododendrons* is of greater urgency than has been previously acknowledge in China. Biodivers. Conserv..

[5] Liu D, Chang Y, Ma Y (2020). Unclear resource background seriously restricts biodiversity conservation of *Rhododendron* in China. Plant Sci. J..

[6] Gibbs, D., Chamberlain, D. & Argent, G. *The Red List of Rhododendrons*. (Botanic Gardens Conservation International, 2011).

[7] Zhang C, Feng B (1996). Investigation of resources condition and growth regularity of *Rhododendron vialii*. Guihaia.

[8] Sun W. *List of Yunnan protected plant species with extremely small populations*. (Yunnan Science and Technology Press, 2021).

[9] Yunnan Administration of Forestry and Grassland, Yunan Department of Agriculture and Rural Affairs & Yunnan Department of Science and Technology. *Plan for Rescuing and Protecting Plant Species with Extremely Small Populations in Yunan Province (2021*–*2030)*. The China government released document (2022).

[10] Gao L, Zhang C, Li D, Wu D (2004). Chromosome numbers of some species of *Rhododeodron*, subgen. Azaleastrum. Acta Bot. Yunnanica.

[11] Simão FA, Waterhouse RM, Ioannidis P, Kriventseva EV, Zdobnov EM (2015). BUSCO: assessing genome assembly and annotation completeness with single-copy orthologs. Bioinformatics.

[12] Doyle J, Doyle JL (1987). A rapid DNA isolation procedure for small quantities of fresh leaf tissue. Phytochem Bull..

[13] Wenger AM (2019). Accurate circular consensus long-read sequencing improves variant detection and assembly of a human genome. Nat. Biotechnol..

[14] Belton JM (2012). Hi-C: a comprehensive technique to capture the conformation of genomes. Methods.

[15] Chen S, Zhou Y, Chen Y, Gu J (2018). fastp: an ultra-fast all-in-one FASTQ preprocessor. Bioinformatics.

[16] Marcais G, Kingsford C (2011). A fast, lock-free approach for efficient parallel counting of occurrences of k-mers. Bioinformatics.

[17] Liu, B. et al. Estimation of genomic characteristics by analyzing k-mer frequency in *de novo* genome projects. Preprint at https://arxiv.org/abs/1308.2012 (2013).

[18] Cheng H, Concepcion GT, Feng X, Zhang H, Li H (2021). Haplotype-resolved de novo assembly using phased assembly graphs with hifiasm. Nat. Methods.

[19] Durand NC (2016). Juicer provides a one-click system for analyzing loop-resolution Hi-C experiments. Cell Syst..

[20] Dudchenko O (2017). De novo assembly of the Aedes aegypti genome using Hi-C yields chromosome-length scaffolds. Science.

[21] Durand NC (2016). Juicebox provides a visualization system for Hi-C contact maps with unlimited zoom. Cell Syst..

[22] Xu M (2020). TGS-GapCloser: A fast and accurate gap closer for large genomes with low coverage of error-prone long reads. GigaScience.

[23] Li H (2018). Minimap2: pairwise alignment for nucleotide sequences. Bioinformatics.

[24] Jin J (2020). GetOrganelle: a fast and versatile toolkit for accurate de novo assembly of organelle genomes. Genome Biol..

[25] Hu J, Fan J, Sun Z, Liu S (2019). NextPolish: a fast and efficient genome polishing tool for long read assembly. Bioinformatics.

[26] Pryszcz LP, Toni G (2016). Redundans: an assembly pipeline for highly heterozygous genomes. Nucleic Acids Res..

[27] Ou S (2019). Benchmarking transposable element annotation methods for creation of a streamlined, comprehensive pipeline. Genome Biol..

[28] Tarailo‐Graovac M, Chen N (2009). Using RepeatMasker to identify repetitive elements in genomic sequences. Curr. Protoc. Bioinformatics.

[29] (2021). NCBI Sequence Read Archive.

[30] Grabherr MG (2011). Full-length transcriptome assembly from RNA-Seq data without a reference genome. Nat. Biotechnol..

[31] Kim D, Langmead B, Salzberg SL (2015). HISAT: a fast spliced aligner with low memory requirements. Nat. Methods.

[32] Pertea M (2015). StringTie enables improved reconstruction of a transcriptome from RNA-seq reads. Nat. Biotechnol..

[33] Haas BJ (2003). Improving the Arabidopsis genome annotation using maximal transcript alignment assemblies. Nucleic Acids Res..

[34] Ma H (2021). Chromosome-level genome assembly and population genetic analysis of a critically endangered rhododendron provide insights into its conservation. Plant J..

[35] Zhou G (2022). Chromosome-scale genome assembly of *Rhododendron molle* provides insights into its evolution and terpenoid biosynthesis. BMC Plant Biol..

[36] Zhang L (2017). The draft genome assembly of *Rhododendron delavayi* Franch. var. delavayi. Gigascience.

[37] Yang F (2020). Chromosome-level genome assembly of a parent species of widely cultivated azaleas. Nat. Commun..

[38] Soza VL (2019). The *Rhododendron* genome and chromosomal organization provide insight into shared whole-genome duplications across the heath family (Ericaceae). Genome Biol. Evol..

[39] Wang X (2021). High-quality evergreen azalea genome reveals tandem duplication-facilitated low-altitude adaptability and floral scent evolution. Plant Biotechnol J..

[40] Zhou X (2022). The chromosome-scale genome assembly, annotation and evolution of *Rhododendron henanense* subsp. *lingbaoense*. Mol. Ecol. Resour..

[41] Diaz-Garcia L (2021). Chromosome-Level Genome Assembly of the American Cranberry (*Vaccinium macrocarpon* Ait.) and Its Wild Relative *Vaccinium microcarpum*. Front. Plant Sci..

[42] Wu H (2019). A high-quality *Actinidia chinensis* (kiwifruit) genome. Hortic. Res..

[43] Zhang X (2021). Haplotype-resolved genome assembly provides insights into evolutionary history of the tea plant *Camellia sinensis*. Nat. Genet..

[44] Kang M (2021). A chromosome-level *Camptotheca acuminata* genome assembly provides insights into the evolutionary origin of camptothecin biosynthesis. Nat. Commun..

[45] Denoeud F (2014). The coffee genome provides insight into the convergent evolution of caffeine biosynthesis. Science.

[46] The French–Italian Public Consortium for Grapevine Genome Characterization (2007). The grapevine genome sequence suggests ancestral hexaploidization in major angiosperm phyla. Nature.

[47] Stanke M, Diekhans M, Baertsch RD, Haussler D (2008). Using native and syntenically mapped cDNA alignments to improve *de novo* gene finding. Bioinformatics.

[48] Cantarel BL (2008). MAKER: an easy-to-use annotation pipeline designed for emerging model organism genomes. Genome Res..

[49] Slater GS, Birney S (2005). Automated generation of heuristics for biological sequence comparison. BMC Bioinformatics.

[50] Brian J (2008). Automated eukaryotic gene structure annotation using EVidenceModeler and the Program to Assemble Spliced Alignments. Genome Biol..

[51] Zhang R (2022). TEsorter: an accurate and fast method to classify LTR-retrotransposons in plant genomes. Hortic. Res..

[52] Chan PP, Lin BY, Mar AJ, Lowe TM (2021). tRNAscan-SE 2.0: improved detection and functional classification of transfer RNA genes. Nucleic Acids Res..

[53] Seemann T. *BAsic Rapid Ribosomal RNA Predictor*. https://github.com/tseemann/barrnap (2018).

[54] Nawrocki EP (2015). Rfam 12.0: updates to the RNA families database. Nucleic Acids Res..

[55] Huerta-Cepas J (2017). Fast Genome-Wide Functional Annotation through Orthology Assignment by eggNOG-Mapper. Mol. Biol. Evol..

[56] Buchfink B, Xie C, Huson DH (2015). Fast and sensitive protein alignment using DIAMOND. Nat. Methods.

[57] Jones P (2014). InterProScan 5: genome-scale protein function classification. Bioinformatics.

[58] Marçais G (2018). MUMmer4: A fast and versatile genome alignment system. PLoS Comput. Biol..

[59] Goel M, Sun H, Jiao W, Schneeberger K (2019). SyRI: finding genomic rearrangements and local sequence differences from whole-genome assemblies. Genome Biol..

[60] Chen M (2021). Genome Warehouse: A Public Repository Housing Genome-scale Data. Genom. Proteom. Bioinfo..

[61] Database Resources of the National Genomics Data Center (2022). China National Center for Bioinformation in 2022. Nucleic Acids Res..

[62] (2023). NGDC Genome Sequence Archive.

[63] (2023). NGDC Genome Sequence Archive.

[64] (2023). NGDC Genome Sequence Archive.

[65] (2023). NGDC Genome Sequence Archive.

[66] (2023). NGDC Genome Warehouse.

[67] (2023). NCBI Sequence Read Archive.

[68] (2023). NCBI Sequence Read Archive.

[69] (2023). NCBI Sequence Read Archive.

[70] (2023). NCBI Sequence Read Archive.

[71] (2023). NCBI Assembly.

[72] (2023). NCBI Assembly.

[73] Li, H. Aligning sequence reads, clone sequences and assembly contigs with BWA-MEM. Preprint at https://arxiv.org/abs/1303.3997 (2013).

[74] Vagheesh N (2016). BCFtools/RoH: a hidden Markov model approach for detecting autozygosity from next-generation sequencing data. Bioinformatics.

