# Supplementary material for: A haplotype-resolved genome assembly of Rhododendron vialii based on PacBio HiFi reads and Hi-C data
Source: Sci Data. 2023 Jul 12;10:451. doi: 10.1038/s41597-023-02362-1 (PMC10338486; doi:10.1038/s41597-023-02362-1)
Supplement: Supplementary file 1 — Supplementary Table [file 41597_2023_2362_MOESM1_ESM.docx]

**Supplementary Tables 1–9**

**Supplementary Table 1.** Statistics of the short-read data on DNBSEQ-T7 platform.

**Supplementary Table 2.** WGS-PacBio sequencing statistics.

**Supplementary Table 3.** Hi-C sequencing statistics.

**Supplementary Table 4.** Iso-Seq data statistics.

**Supplementary Table 5.** Summary of the basic features of the estimated genome.

**Supplementary Table 6.** Statistics of chromosomal level assembly of *R*. *vialii*.

**Supplementary Table 7.** Repeat annotations of the *R*. *vialii* genome assembly.

**Supplementary Table 8.** Summary of the transcriptome assemblies.

**Supplementary Table 9.** Functional annotation of predicted genes in *R*. *vialii* genome.

**Supplementary Table 1.** Statistics of the short-read data on DNBSEQ-T7 platform.

| **Raw reads(M)** | **Raw bases(G)** | **Raw Q20(G)** | **Raw Q30(G)** | **Clean reads(M)** | **Clean bases(G)** | **Clean Q20(G)** | **Clean Q30(G)** | **Average length(bp)** |
| --- | --- | --- | --- | --- | --- | --- | --- | --- |
| 649.241 | 97.386 | 94.915 (97.46%) | 89.942 (92.36%) | 649.129 (99.98%) | 95.120 (97.67%) | 92.797 (97.56%) | 89.942 (92.47%) | 146.5 |

**Supplementary Table 2.** WGS-PacBio sequencing statistics.

| **Total bases (bp)** | **GC content** | **A (bp)** | | **T (bp)** | | **G (bp)** | | **C (bp)** | | |
| --- | --- | --- | --- | --- | --- | --- | --- | --- | --- | --- |
| 32,888,038,407 | 39.61(%) | 9,936,184,840 (30.21%) | | 9,925,462,793 (30.18%) | | 6,520,231,334 (19.83%) | | 6,506,159,440 (19.78%) | | |
| **Total reads** | **Max. (bp)** | **Mean (bp)** | **N10 (bp)** | **N50 (bp)** | **N90 (bp)** | **Min.(bp)** | **Median (bp)** | **L10** | **L50** | **L90** |
| 1,799,266 | 87,469 | 18,278 | 24,731 | 18,109 | 14,708 | 519 | 17,379 | 121,480 | 758,479 | 1,566,125 |

**Supplementary Table 3.** Hi-C sequencing statistics.

| **Reads (M)** | **Bases (G)** | **Q20 (G)** | **Q30 (G)** | **Average length (bp)** |
| --- | --- | --- | --- | --- |
| 500.383 | 75.057 | 73.805 (98.3%) | 70.364 (93.7%) | 150 |

**Supplementary Table 4.** Iso-Seq data statistics.

| **Total bases (bp)** | **GC content** | **A (bp)** | | **T (bp)** | | **G (bp)** | | **C (bp)** | | |
| --- | --- | --- | --- | --- | --- | --- | --- | --- | --- | --- |
| 10,361,844,440 | 44.40 (%) | 2,857,016,363 (27.57%) | | 2,903,843,167 (28.02%) | | 2,308,401,426 (22.28%) | | 2,292,583,484 (22.13%) | | |
| **Total reads** | **Max. (bp)** | **Mean (bp)** | **N10 (bp)** | **N50 (bp)** | **N90 (bp)** | **Min.(bp)** | **Median (bp)** | **L10** | **L50** | **L90** |
| 10,890,369 | 29,673 | 951 | 2,597 | 1,190 | 500 | 86 | 777 | 309,570 | 2,804,642 | 8,011,525 |

**Supplementary Table 5.** Summary of the basic features of the estimated genome.

| **K-mer** | **K-mer number** | **K-mer depth** | **Genome size (Mb)** | **Coverage** | **Heterozygous ratio (%)** | **Duplication ratio (%)** |
| --- | --- | --- | --- | --- | --- | --- |
| 19 | 75,123,904,907 | 142.92 | 525.63 | 180.91 | 0.89 | 43.47 |

**Supplementary Table 6.** Statistics of chromosomal level assembly of *R*. *vialii*.

| **Chr ID** | **Length (bp)** | **Contig number** | **Gap number** | **Gap length (bp)** |
| --- | --- | --- | --- | --- |
| chr01a | 49,061,468 | 1 | 0 | 0 |
| chr01b | 45,509,457 | 2 | 1 | 100 |
| chr02a | 47,677,579 | 2 | 1 | 100 |
| chr02b | 46,758,727 | 2 | 1 | 99 |
| chr03a | 36,343,144 | 1 | 0 | 0 |
| chr03b | 35,747,676 | 2 | 1 | 100 |
| chr04a | 44,228,087 | 1 | 0 | 0 |
| chr04b | 44,137,505 | 1 | 0 | 0 |
| chr05a | 43,106,196 | 2 | 1 | 95 |
| chr05b | 42,149,085 | 2 | 1 | 100 |
| chr06a | 43,937,226 | 2 | 1 | 100 |
| chr06b | 42,443,326 | 1 | 0 | 0 |
| chr07a | 42,050,137 | 1 | 0 | 0 |
| chr07b | 41,813,812 | 2 | 1 | 98 |
| chr08a | 36,345,595 | 2 | 1 | 100 |
| chr08b | 36,759,256 | 1 | 0 | 0 |
| chr09a | 40,475,278 | 2 | 1 | 100 |
| chr09b | 38,702,740 | 2 | 1 | 100 |
| chr10a | 39,676,699 | 1 | 0 | 0 |
| chr10b | 38,161,561 | 2 | 1 | 100 |
| chr11a | 39,472,952 | 1 | 0 | 0 |
| chr11b | 39,425,882 | 1 | 0 | 0 |
| chr12a | 35,586,280 | 1 | 0 | 0 |
| chr12b | 35,674,509 | 1 | 0 | 0 |
| chr13a | 34,773,050 | 1 | 0 | 0 |
| chr13b | 34,698,656 | 1 | 0 | 0 |
| Total | 1,054,715,883 (99.92%) | | | |
| Pt | 177,205 (0.06%) | 1 | 0 | 0 |
| Mt | 679,428 (0.02%) | 1 | 0 | 0 |
| tg | 0 | — | — | — |

**Supplementary Table 7.** Repeat annotations of the *R*. *vialii* genome assembly.

| **Type** | **Superfamily** | **Number** | **Length (bp)** | **Percent (%)** | **Mean length (bp)** |
| --- | --- | --- | --- | --- | --- |
| LTR | Copia | 60,554 | 40,691,620 | 3.85 | 671.99 |
|  | Gypsy | 156,510 | 177,310,113 | 16.8 | 1,132.9 |
|  | Retrovirus | 226 | 72,794 | 0.01 | 322.1 |
|  | Unknown | 133,824 | 68,801,299 | 6.52 | 514.12 |
|  | Total | 351,114 | 286,875,826 | 27.18 | 817.04 |
| LINE |  | 2,143 | 1,299,831 | 0.12 | 606.55 |
| DNA | Helitron | 186,055 | 34,735,710 | 3.29 | 186.7 |
| TIR | EnSpm_CACTA | 74,742 | 17,758,067 | 1.68 | 237.59 |
|  | MuDR_Mutator | 162,113 | 89,707,702 | 8.5 | 553.37 |
|  | PIF_Harbinger | 78,451 | 17,486,825 | 1.66 | 222.9 |
|  | Tc1_Mariner | 177,153 | 32,359,608 | 3.07 | 182.66 |
|  | hAT | 95,937 | 24,824,928 | 2.35 | 258.76 |
|  | Total | 588,396 | 182,137,130 | 17.25 | 309.55 |
| Unknown |  | 97,622 | 33,132,547 | 3.14 | 339.4 |
| Simple_repeat |  | 272,371 | 11,100,174 | 1.05 | 40.75 |
| Low_complexity |  | 36,332 | 1,730,593 | 0.16 | 47.63 |
| Polinton |  | 175 | 49,058 | 0 | 280.33 |
| Total |  | 1,534,208 | 551,060,869 | 52.19 | 358.84 |

**Supplementary Table 8.** Summary of the transcriptome assemblies.

| **Pipelines** | **Number of genes** | **Length of total genes (bp)** | **Average gene length (bp)** | **Number of transcripts** | **Length of total transcript (bp)** | **Average transcript length (bp)** | **Length of N50 transcript (bp)** |
| --- | --- | --- | --- | --- | --- | --- | --- |
| Trinity *de* *novo* (RNA-Seq) | 37,674 | 30,038,842 | 797.34 | 59,147 | 54,473,203 | 920.98 | 1,453 |
| Hisat2 + StringTie (RNA-Seq) | 51,137 | 63,189,560 | 1,235.69 | 69,505 | 91,843,822 | 1,321.40 | 1,759 |
| Minimap2 + StringTie (Iso-Seq) | 47,770 | 116,858,468 | 2,446.27 | 82,341 | 199,771,725 | 2,426.15 | 2,889 |
| Merged by PASA | 57,486 | 122,345,906 | 2,128.27 | 114,558 | 240,081,399 | 2,095.72 | 2,680 |

**Supplementary Table 9.** Functional annotation of predicted genes in *R*. *vialii* genome.

| **Database** | **Gene number** | **Percentage (%)** |
| --- | --- | --- |
| All | 60,929 | 100 |
| GO | 25,613 | 42.04 |
| KEEG_Pathway | 15,039 | 24.68 |
| KEEG_KO | 24,439 | 40.11 |
| eggNOG | 50,042 | 82.14 |
| COG | 53,285 | 87.46 |
| EC | 10,836 | 17.79 |
| Swiss_Prot | 39,487 | 64.81 |
| TrEMBL | 55,377 | 90.89 |
| NR | 52,866 | 86.77 |
| *A*. *thaliana* | 47,000 | 77.14 |
| Pfam | 45,777 | 75.14 |
| CDD | 18,236 | 29.93 |
| PRINTS | 7,662 | 12.58 |
| Interpro | 48,177 | 79.07 |
| Phobius | 20,170 | 33.11 |
| Gene3D | 38,237 | 62.76 |
| SUPERFAMILY | 35,520 | 58.30 |
| TIGRFAM | 5,755 | 9.45 |
| Coils | 8,926 | 14.65 |
| SMART | 16,974 | 27.86 |
| Unannotated | 3,704 | 6.08 |
