# Supplementary material for: A haplotype-resolved genome assembly of Rhododendron vialii based on PacBio HiFi reads and Hi-C data
Source: Sci Data. 2023 Jul 12;10:451. doi: 10.1038/s41597-023-02362-1 (PMC10338486; doi:10.1038/s41597-023-02362-1)
Supplement: Supplementary file 2 — Supplementary Figure [file 41597_2023_2362_MOESM2_ESM.docx]

**Supplementary Figures 1–3**

**Supplementary Figure 1.** The depth-distribution of 19-mers.

**Supplementary Figure 2.** Dot-plot of synteny blocks between *Rhododendron* *vialii* and *R*. *griersonianum* (a) and the two haplotypes within *R*. *vialii* (b).

**Supplementary Figure 3.** Structural variation between the two haplotypes of *R*. *vialii*.

**

Supplementary Figure 1.** The depth-distribution of 19-mers.





**Supplementary Figure 2.** Dot-plot of synteny blocks between *Rhododendron* *vialii* and *R*. *griersonianum* (a) and the two haplotypes within *R*. *vialii* (b).


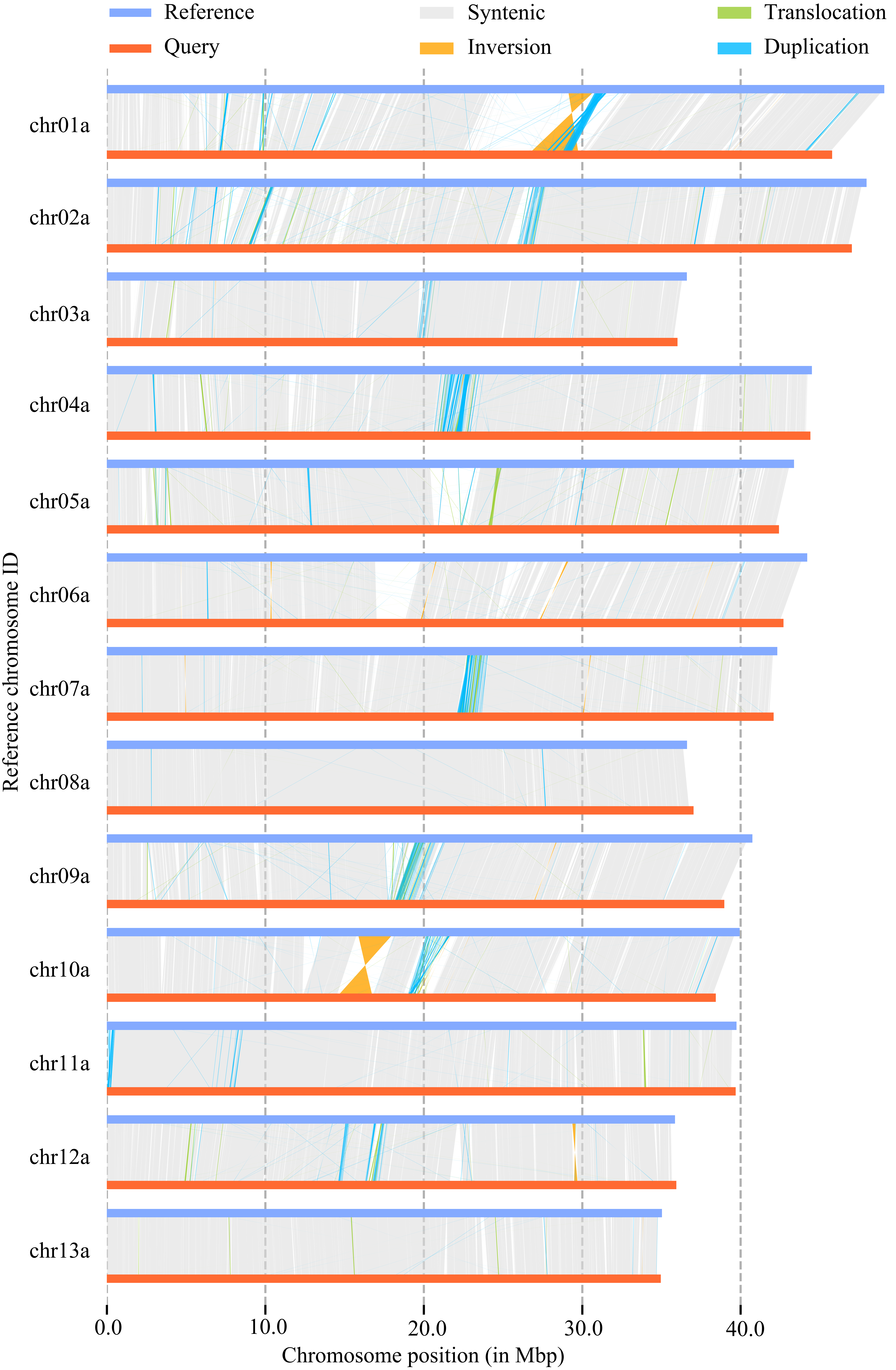


**Supplementary Figure 3.** Structural variation between the two haplotypes of *R*. *vialii*.
